# Genetic and Physiologic Dissection of the Vertebrate Cardiac Conduction System

**DOI:** 10.1371/journal.pbio.0060109

**Published:** 2008-05-13

**Authors:** Neil C Chi, Robin M Shaw, Benno Jungblut, Jan Huisken, Tania Ferrer, Rima Arnaout, Ian Scott, Dimitris Beis, Tong Xiao, Herwig Baier, Lily Y Jan, Martin Tristani-Firouzi, Didier Y. R Stainier

**Affiliations:** 1 Department of Biochemistry and Biophysics and Programs in Developmental Biology, Genetics, and Human Genetics, University of California San Francisco, San Francisco, California, United States of America; 2 Cardiovascular Research Institute, University of California San Francisco, San Francisco, California, United States of America; 3 Department of Medicine, University of California San Francisco, San Francisco, California, United States of America; 4 Department of Physiology, University of California San Francisco, San Francisco, California, United States of America; 5 Howard Hughes Medical Institute, University of California San Francisco, San Francisco, California, United States of America; 6 Department of Pediatrics, University of Utah, Salt Lake City, Utah, United States of America; 7 Nora Eccles Harrison Cardiovascular Research and Training Institute, University of Utah, Salt Lake City, Utah, United States of America; 8 Harvard Medical School, Boston, Massachusetts, United States of America; 9 Program in Developmental and Stem Cell Biology, The Hospital for Sick Children, and Department of Molecular Genetics, University of Toronto, Toronto, Ontario, Canada; 10 Developmental Biology, Biomedical Research Foundation, Academy of Athens, Athens, Greece; 11 Department of Physiology, University of California San Francisco, San Francisco, California, United States of America; 12 Programs in Neuroscience, Genetics, Human Genetics, and Developmental Biology, University of California San Francisco, San Francisco, California, United States of America; Duke University, United States of America

## Abstract

Vertebrate hearts depend on highly specialized cardiomyocytes that form the cardiac conduction system (CCS) to coordinate chamber contraction and drive blood efficiently and unidirectionally throughout the organism. Defects in this specialized wiring system can lead to syncope and sudden cardiac death. Thus, a greater understanding of cardiac conduction development may help to prevent these devastating clinical outcomes. Utilizing a cardiac-specific fluorescent calcium indicator zebrafish transgenic line, *Tg*(*cmlc2*:*gCaMP*)*^s878^*, that allows for in vivo optical mapping analysis in intact animals, we identified and analyzed four distinct stages of cardiac conduction development that correspond to cellular and anatomical changes of the developing heart. Additionally, we observed that epigenetic factors, such as hemodynamic flow and contraction, regulate the fast conduction network of this specialized electrical system. To identify novel regulators of the CCS, we designed and performed a new, physiology-based, forward genetic screen and identified for the first time, to our knowledge, 17 conduction-specific mutations. Positional cloning of *hobgoblin^s634^* revealed that *tcf2*, a homeobox transcription factor gene involved in mature onset diabetes of the young and familial glomerulocystic kidney disease, also regulates conduction between the atrium and the ventricle. The combination of the *Tg*(*cmlc2*:*gCaMP*)*^s878^* line/in vivo optical mapping technique and characterization of cardiac conduction mutants provides a novel multidisciplinary approach to further understand the molecular determinants of the vertebrate CCS.

## Author Summary

Aberrant electrical activity of the heart, otherwise known as cardiac arrhythmia, may disrupt heart contractions, leading to loss of consciousness and sudden death. Every year, approximately 450,000 individuals in the United States die suddenly from this event. Currently, the only proven preventive therapy for sudden cardiac death is the automatic implantable cardioverter defibrillator, which carries a significant burden and cost to the patient. Greater understanding of the cardiac conduction system, which coordinates rhythmic beating of the heart, may lead to novel and safer therapeutic options for these patients. Working with zebrafish, a productive model system for understanding human disease, we have developed a cardiac-specific fluorescent calcium indicator zebrafish transgenic line to analyze the formation of the cardiac conduction system. Using this fluorescent transgenic line, we have observed four distinct physiologic cardiac conduction stages that correspond to cellular and anatomic changes of the developing heart. Furthermore, we have designed and performed a new, physiology-based, forward genetic screen to identify cardiac conduction mutants that would have escaped discovery in previous screens. Overall, these studies may prove rewarding toward developing therapeutic options aimed at maintaining and/or improving overall cardiac health.

## Abstract 3

By characterizing the genetic underpinnings of the vertebrate cardiac conduction system, a new study sheds light on the mechanisms underlying cardiac arrhythmias, which cause the sudden death of hundreds of thousands of people each year.

## Introduction

Vertebrate hearts have evolved into multichambered structures requiring coordinated beating of their chambers to achieve antegrade blood flow throughout the organism. Unidirectional blood flow is achieved through two specialized structures that are unique to vertebrates: cardiac valves and the specialized cardiac conduction system (CCS). In the adult heart, the initial electrical impulses are generated in the slow pacemaker sino-atrial (SA) node and then propagated across the atrium. This electrical impulse is delayed at the atrioventricular (AV) boundary through specialized slow conducting AV node cardiomyocytes. After the delay at the AV node, electrical propagation travels rapidly through the fast conduction network comprised of the His-Purkinje system, which coordinates ventricular activation to occur from the apex to the base of the heart. This apex-to-base activation allows for efficient ejection of blood from the ventricles into the outflow tracts (OFTs) at the base of the heart [1].

Despite extensive knowledge of the anatomy and physiology of the adult vertebrate CCS, the cellular and molecular events that govern the development of this specialized tissue remain unclear. Lineage tracing studies have revealed that the CCS is derived from cardiomyocyte progenitors [2,3]. Myocardial factors that regulate the specification of the CCS include Nkx2.5 and Tbx5 [2,4]. Loss of either transcriptional regulator leads to defects in the maturation and maintenance of the AV conduction system and subsequent AV heart block and bundle branch block.

Additional studies have revealed the requirement of the endocardium for cardiomyocyte specification to form the fast conduction network within the ventricle [5–7]. Secreted factors from endocardial as well as other cardiac endothelial cells, such as Endothelin 1 and Neuregulin, are able to induce cardiac conduction markers in cultured embryonic cardiomyocytes and cultured hearts [7–9]. Furthermore, hemodynamic changes regulate the secretion of Endothelin 1 from endocardial cells, thereby affecting the development of the fast conduction pathway [6]. More recently, the role of the endocardium for the development of AV conduction delay has been investigated further using the zebrafish *cloche* mutant [5], which lacks endothelial cells among other defects [10]. That study concluded that Neuregulin but not Endothelin 1 is required for the induction of AV conduction delay.

Optical mapping of cardiac excitation using voltage- and calcium-sensitive dyes has allowed the spatiotemporal analysis of electrical excitation wave dynamics, not only advancing our understanding of the electrical activity during cardiac arrhythmias but also allowing for further analysis of CCS development [11]. However, the use of voltage- and calcium-sensitive dyes is associated with serious shortcomings, including a lack of cellular targeting, limited live animal experimentation, the need for physical loading of these indicators into cells, and cellular toxicity. To circumvent these problems, fluorescent calcium indicator proteins have begun to replace voltage- and calcium-sensitive dyes for physiologic in vivo analysis of tissue/organ electrical activity in different animal model systems including fly and mouse [12–14]. Yet, optical mapping of mouse hearts is currently limited due to explantation for ex vivo analysis. Thus, we have taken advantage of the external fertilization and translucency of zebrafish embryos to create a cardiac-specific fluorescent calcium indicator transgenic line, *Tg*(*cmlc2*:*gCaMP*)*^s878^*, to perform in vivo optical mapping analyses throughout the stages of heart development.

Here we describe a multidisciplinary approach using the zebrafish toward understanding CCS development. Utilizing the *Tg*(*cmlc2*:*gCaMP*)*^s878^* optical mapping system, we identified four distinct physiologic developmental stages of the CCS that correspond to cellular and anatomical changes of the developing zebrafish heart. (1) Initially, a linear conduction travels across the heart tube from the sinus venosus to the OFT (20–24 hours postfertilization (hpf)); (2) next, a significant AV conduction delay develops during cardiac chamber formation (36–48 hpf); (3) as the heart loops and develops ventricular trabeculations (72–96 hpf), an immature fast conduction network develops within the ventricle; (4) finally, this fast conduction network fully matures to an apex-to-base activation pattern when the ventricular apex has formed.

Furthermore, to identify regulators of CCS development, we performed a diploid ethylnitrosourea (ENU) mutagenesis screen and recovered several novel as well as known cardiovascular conduction/rhythm mutants, which we have analyzed using in vivo optical imaging techniques and classified according to the affected physiologic developmental stage of the CCS. Positional cloning of *hobgoblin* (*hob*), a novel mutant with AV heart block, reveals that *tcf2*, a homeobox transcription factor gene involved in mature onset diabetes of the young, also regulates conduction between the atrium and the ventricle. Thus, these detailed electrophysiologic and genetic analyses of wild-type and mutant hearts provide further insights into the development of the vertebrate CCS and will lead to a better understanding of the pathogenesis of cardiac arrhythmias.

## Results

### Optical Mapping of *Tg*(*cmlc2*:*gCaMP*)*^s878^* Hearts Reveals Distinct Developmental Stages of the Vertebrate CCS

Previous studies have utilized calcium green, a calcium-sensitive fluorescent indicator, in zebrafish hearts to observe cardiac conduction up to 48 hpf [5,15]. However, because loading these hearts and/or embryos with calcium green is technically cumbersome and is only temporary, we created a zebrafish transgenic line *Tg*(*cmlc2*:*gCaMP*)*^s878^* that specifically expresses *gCaMP*, a genetically encoded calcium reporter based on a circular permutation of green fluorescent protein (GFP) [16], at all developmental stages in the heart, using the cardiac-specific promoter *cmlc2* [17] (Figure S1).

**Figure 1:**
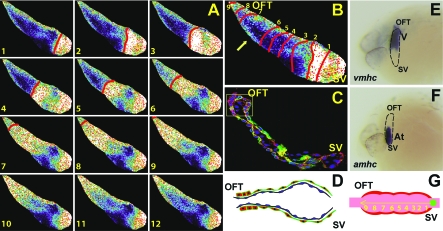
Cellular and In Vivo Electrophysiologic Analysis of 24 hpf Wild-Type Hearts (A) Sequential calcium activation images of a 24 hpf wild-type *Tg*(*cmlc2*:*gCaMP*)*^s878^* heart during a single cycle in a live zebrafish embryo. (B) A 24 hpf optical map of calcium excitation represented by isochronal lines every 60 ms. Slow linear conduction with no significant delays is observed across the 24 hpf heart tube with increased conduction velocity over the presumptive ventricle (arrow). (C) *Tg*(*cmlc2*:*eGFP-ras*)*^s883^* (green) embryos at 24 hpf are stained with rhodamine phalloidin (red) and TOPRO (dark blue). The heart is a linear tube, and cardiomyocytes uniformly have a squamous shape except at the OFT (yellow box) where they have become cuboidal. (D) Schematic representation of the heart shown in (C). Myocardium is in red. Endocardium is in blue. (E and F) In situ analysis of 24 hpf embryos reveals molecular chamber specification within the heart tube: *atrial myosin heavy chain*, *amh*c, and *ventricular myosin heavy chain*, *vmhc*. Lateral views. (G) Schematic representation of conduction in 24 hpf heart. Numbers indicate temporal sequence of calcium activation in the heart. Yellow arrow indicates direction of cardiac conduction. Green circle indicates slow conduction pathway/pacemaker activity. SV, sinus venosus; V, ventricle; At, atrium.

Because cardiac contraction and blood flow begins at the linear heart tube (LHT) stage, we initiated our optical mapping studies on the LHT of 24 hpf *Tg*(*cmlc2*:*gCaMP*)*^s878^* embryos. These experiments revealed that conduction travels unidirectionally in a relatively slow and linear pattern without significant pauses from the sinus venosus to the OFT, suggesting the presence of a functional SA node pacemaker activity (Figure 1A and 1B and Video S1). Additionally, an acceleration of conduction was observed within the OFT half of the heart (Figure 1B, arrow).

Utilizing the *Tg*(*cmlc2*:*eGFP-ras*)*^s883^* line (Jungblut B, Munson C, Huisken J, Trinh L, Stainier D, unpublished data ), which outlines individual cardiomyocytes with membrane-bound GFP, confocal microscopy was performed to analyze further the cellular characteristics of the LHT. Despite displaying atrial and ventricular molecular changes [18] (Figure 1E and 1F), 24 hpf cardiomyocytes maintain a nearly uniform squamous morphology on cross-sectional analysis (Figure 1C). However, we observed a small subpopulation of cuboidal cardiomyocytes near the OFT (Figure 1C, box). These cuboidal cardiomyocytes correlate with the acceleration of conduction observed in the optical mapping of the LHT.

### Cellular Changes of the Atrial, Ventricular, and AV Myocardial Cells Occur at 36–48 hpf and Correlate with AV Conduction Delay

**Figure 2:**
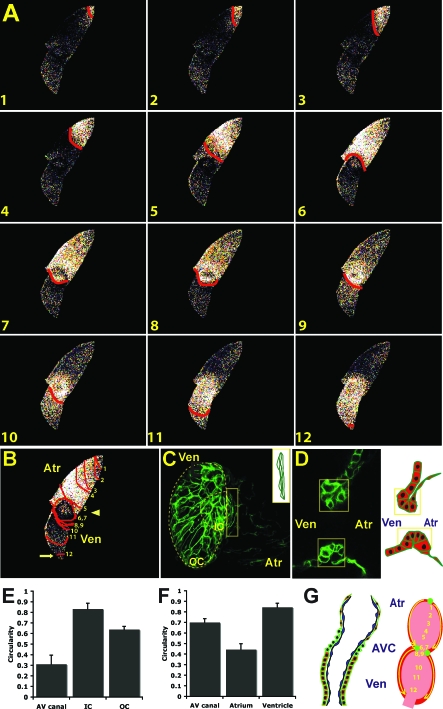
Development of AV Myocardium Results in AV Conduction Delay and Development of the Central CCS (A) Sequential calcium activation images of a 48 hpf wild-type *Tg*(*cmlc2*:*gCaMP*)*^s878^* heart during a single cycle in a live zebrafish embryo. (B) The 48 hpf optical maps of calcium excitation represented by isochronal lines every 60 ms. Significant conduction delay was observed at the AV junction (arrowhead) and OFT (arrow). (C) Surface and (D) cross-sectional analyses of *Tg*(*cmlc2*:*eGFP-ras*)*^s883^* hearts at 48 hpf reveal distinct cellular morphologies and orientations among atrial, ventricular, and AV (yellow boxes) cardiomyocytes. Schematic illustration of AV canal region to the right. OC and IC are outlined on projection of ventricle in (C). (E and F) Bar graphs represent cell morphology/circularity measurements of *Tg*(*cmlc2*:*eGFP-ras*)*^s883^* hearts at 48 hpf: (E) surface of cardiomyocytes from the OC and IC of the ventricle as well as the AV myocardium and (F) atrial, ventricular, and AV canal cardiomyocytes on cross-sectional analysis. (G) Schematic representation of a 48 hpf heart. Individual cardiomyocytes are outlined in green. Endocardium is in blue. Numbers indicate temporal sequence of calcium activation in the heart. Yellow arrows indicate direction of cardiac conduction. Green circles indicate slow conduction pathway/pacemaker and AV conduction delay. Atr, atrium; Ven, ventricle; AVC, AV canal.

By 36–48 hpf, the zebrafish embryonic heart has developed a distinct AV canal that separates the cardiac chambers (Figure 2C). Calcium activation travels from the sinus venosus across the atrium to the AV canal (Figure 2A and 2B). At the AV canal, a dramatic slowing of the calcium activation wave was observed as illustrated by an increased number of isochronal lines at the AV boundary between the atrial and the ventricular chambers (Figure 2B, arrowhead). Subsequently, ventricular calcium activation proceeded from the AV canal, accelerating laterally across the ventricular myocardium to the OFT where significant deceleration of conduction was observed (Figure 2B, arrow, and Video S2). These AV and OFT conduction delays may help to prevent regurgitant blood flow between the atrial and the ventricular chambers as well as between the heart and the bulbus arteriosus, respectively, thus resulting in efficient antegrade blood flow throughout the embryo.

To determine whether cell morphology or orientation may correlate with these conduction velocity differences, we analyzed and measured cardiomyocytes of 48 hpf *Tg*(*cmlc2*:*eGFP-ras*)*^s883^* hearts. On cross-sectional analysis, it appeared that atrial cardiomyocytes had maintained their squamous cell morphology while ventricular cardiomyocytes had become cuboidal (Figure 2D and 2F) and cardiomyocytes at the AV boundary had initiated apical membrane constriction, resulting in cells with a distinct trapezoidal shape (Figure 2D, yellow box). Furthermore, examination of the surface of ventricular cardiomyocytes revealed that outer curvature (OC) cardiomyocytes (the greater, convex curvature of the cardiac chamber) became elongated while inner curvature (IC) cardiomyocytes (the lesser, concave curvature of the cardiac chamber) remained rounded (Figure 2C and 2E), as previously described [19]. Interestingly, cardiomyocytes at the AV boundary also became significantly elongated, forming a ring of cells around the AV canal (Figure 2C, yellow box and inset). The orientation of these AV cardiomyocytes was orthogonal to that of the OC ventricular cardiomyocytes.

**Figure 3:**
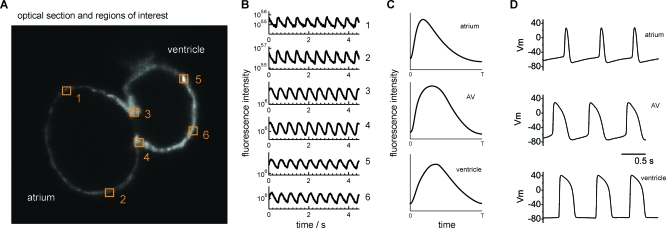
Calcium Transients and APs Confirm That AV Myocardium Has Distinct Electrophysiologic Properties That Distinguish It from Atrium and Ventricle (A) Optical section of 48 hpf *Tg*(*cmlc2*:*gCaMP*)*^s878^* heart. Numbers represent areas where calcium transients for the atrium, ventricle, and AV canal were recorded. (B) Fluorescence intensity of a single pixel from each region was recorded to obtain calcium transients and plotted over time in seconds. All plots are semilogarithmic and identically scaled. (C) Average calcium transient for each cardiac region. (D) The APs from each of these regions were recorded in wild-type explanted 48 hpf hearts using patch pipettes and current clamp techniques. Distinct calcium transients and APs were detected in atrium, AV canal, and ventricle.

To determine whether these different populations of myocardial cells exhibit distinct electrophysiologic properties, calcium transients of the atrium, ventricle, and AV canal were recorded from fluorescence of a single pixel from each region. Distinct calcium transients were recorded from each region of 48 hpf *Tg*(*cmlc2*:*gCaMP*)*^s878^* hearts (Figure 3B and 3C). To further test these findings, action potentials (APs) were recorded in explanted 48 hpf wild-type hearts using patch pipettes and the current clamp technique (Figure 3D). The APs recorded from zebrafish atrium were similar in morphology to those reported in embryonic mammalian myocardium, including spontaneous diastolic depolarization. The APs recorded from zebrafish ventricle were typical of mammalian ventricles, with a flat diastolic phase and an overt systolic plateau phase. The APs recorded from the AV canal were distinguishable from atrial and ventricular APs by the presence of a slow diastolic depolarization phase in the setting of an overt systolic plateau phase. The morphology of the APs recorded from the wild-type AV canal was similar to that reported for the mammalian AV node [20], suggesting that the distinct electrophysiologic properties of AV myocardial cells contribute to the conduction delay between the cardiac chambers.

### Development of the Rapid Ventricular CCS Occurs at 100 hpf when Hearts Develop Ventricular Trabeculation

To achieve efficient ejection of blood from the ventricle to the arterial system, the adult vertebrate ventricle contracts from the apex to the base of the heart (i.e., where the AV canal and OFT reside). This contraction pattern is achieved through the fast cardiac conduction network (the His-Purkinje system), which passes through the ventricular septum in amniotes and allows for apex-to-base conduction across the ventricular myocardium. Previous studies have shown that the fast CCS in chick and mammalian hearts may initially develop along ventricular trabeculae after cardiac looping but prior to ventricular septation [6,21]. To understand further how the fast CCS develops, we analyzed zebrafish hearts at stages after cardiac looping.

**Figure 4:**
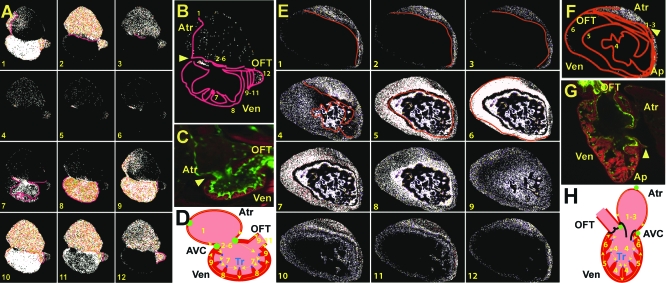
Development of the Fast Specialized Cardiac Conduction Tissue Cellular and electrophysiologic analysis of wild-type *Tg*(*cmlc2*:*gCaMP*)*^s878^* hearts at 100 hpf and 21 dpf. (A and E) Sequential calcium activation images of a 100 hpf (A) *Tg*(*cmlc2*:*gCaMP*)*^s878^* heart and a 21 dpf (E) *Tg*(*cmlc2*:*gCaMP*)*^s878^* ventricle during a single cycle in a live zebrafish. (B and F) The 100 hpf (B) and 21 dpf (F) optical maps of calcium excitation represented by isochronal lines every 60 ms. Ventricular calcium activation initiates along trabeculae at 100 hpf and 21 dpf and breaks through the rest of the myocardium propagating from OC to base at 100 hpf and apex to base at 21 dpf. (C and G) Confocal images of the heart at 100 hpf and 21 dpf. (C) The 100 hpf *Tg*(*flk1*:*EGFP*)*^s843^* (green) larvae stained with rhodamine phalloidin (red) reveal that the hearts have completed cardiac looping and that the ventricles have formed trabeculae.(G) At 21 dpf, the ventricle forms a distinct apex. (D and H) Schematic representation of the hearts shown in (C) and (G). Numbers indicate temporal sequence of calcium activation in the heart. Yellow arrows indicate direction of cardiac conduction. Green circles indicate slow conduction nodes, and blue circles indicate fast conduction pathway. Atr, atrium; Ven, ventricle; AVC, AV canal; Tr, trabeculae.

Using the *Tg*(*flk1*:*eGFP*)*^s843^* line, which marks endocardial cells with green fluorescence [22], we observed that rhodamine-phalloidin–stained 100 hpf zebrafish hearts not only have completed cardiac looping but also have initiated ventricular trabeculation (Figure 4C). By 2–3 weeks postfertilization, the ventricle has developed an apex and increased its trabeculation (Figure 4G).

Optical mapping of 100 hpf *Tg*(*cmlc2*:*gCaMP*)*^s878^* hearts revealed that ventricular conduction has transformed from a primitive linear propagation traveling from the AV canal to the OFT as observed at 48 hpf (Figure 2) to a more complex conduction pattern propagating from the OC of the ventricle to the base/IC. After the AV conduction delay, the earliest ventricular calcium activation was observed along the trabeculae (Figure 4A and 4B, activation sequence 7). Next, calcium activation rapidly proceeded from the trabeculae to the adjacent peripheral cardiomyocytes, leading to conduction from the OC to the base/IC (Figure 4A and 4B, activation sequences 8 and 9, and Video S3). Finally, similar to 48 hpf hearts, ventricular conduction terminated at the OFT where conduction velocity significantly decelerated (Figure 4A and 4B, activation sequences 9–12). Interestingly, trabeculae were not observed within the OFT (Figure 4C), further suggesting that they may be responsible for rapid ventricular conduction.

At 2–3 weeks of life, when the ventricle clearly forms an apex, we observed that the initial conduction along the trabeculae (Figures 4E and 4F, sequence activation 4) breaks through at the apex, resulting in apex-to-base/IC conduction (Figure 4E and 4F, activation sequences 5 and 6). Thus, these results indicate that the fast cardiac conduction pathway develops prior to the formation of the apex, resulting in ventricular conduction from the OC to the base/IC to allow efficient ejection of blood from the heart shortly after cardiac looping.

**Figure 5:**
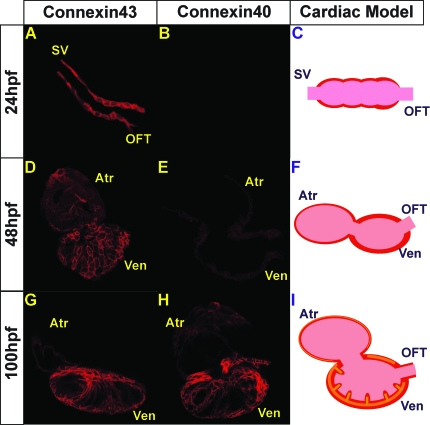
Immunolocalization of Cx40 and Cx43 in Wild-Type Hearts Confocal images of the heart at 24, 48, and 100 hpf, following immunostaining with anti-Cx40 or anti-Cx43 antibodies (red). (A, D, and G) Cx43 immunoreactivity was observed throughout the myocardium at 24, 48, and 100 hpf while (B, E and H) Cx40 immunoreactivity was observed weakly at 48 hpf and strongly at 100 hpf. (C, F and I) Schematic representation of Connexin staining at each developmental stage. Cx43 immunoreactivity is observed throughout the myocardium at all stages and represented in red, and Cx40 immunoreactivity is observed at later cardiac developmental stages and represented in orange.

To understand further the development of the fast CCS, we examined the expression of gap junction proteins responsible for cardiac conduction. *Connexin40* (*Cx40*) is expressed in the atrium and the fast conduction system of mammalian hearts, and loss of this gap junction protein results in reduced cardiac conduction velocity and a predisposition to cardiac arrhythmias [23]. In contrast, *Connexin43* (*Cx43*) is expressed abundantly throughout the atrial and ventricular myocardium but in lower amounts in the fast conduction system [24,25]. Because both *cx40* and *cx43* have been suggested to be expressed in zebrafish hearts [26,27], we performed immunostaining on these hearts with antibodies against Cx40 and Cx43. Cx43 immunostaining is present throughout the heart from 24 hpf onwards (Figure 5A, 5D, and 5G). However, Cx40 immunoreactivity is not observed at 24 hpf, but is detected very weakly at 48 hpf, and strongly by 100 hpf throughout the myocardium (Figure 5B, 5E, and 5H). Thus, Cx40 immunoreactivity is present during the development of the fast CCS.

### Hemodynamic Flow and Contraction Affect the Development of the CCS

**Figure 6:**
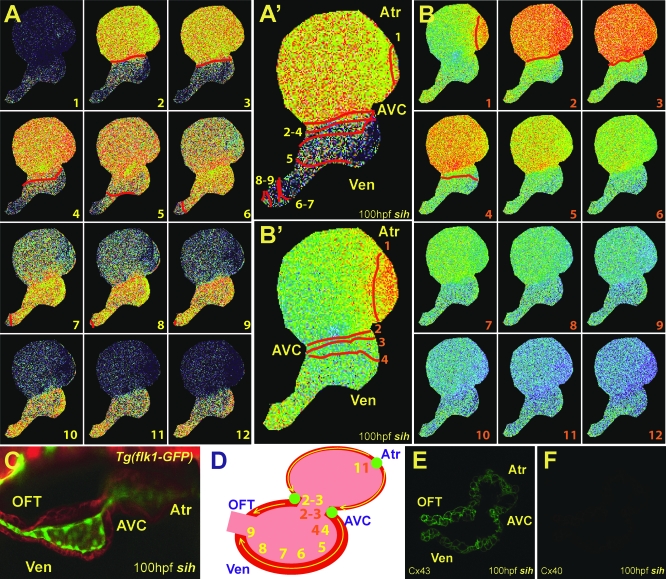
Hemodynamic Flow Is Required for the Development of the Fast CCS (A and B) Sequential calcium activation images of a 100 hpf *Tg*(*cmlc2*:*gCaMP*)*^s878^*; *sih* mutant heart during two different cardiac cycles in a live zebrafish embryo. (A′ and B′) Optical maps of calcium excitation of 100 hpf *sih* mutant hearts represented by isochronal lines every 60 ms. (C) Confocal images of *sih* mutant hearts at 100 hpf. *Tg*(*flk1*:*EGFP*)*^s843^*; *sih* mutant embryos were stained with rhodamine phalloidin (red). The heart has completed cardiac looping; however, the ventricle has failed to form trabeculae. (D) Schematic representation of the heart shown in (C). Yellow and orange numbers indicate sequential calcium activation in *sih* mutant hearts from (A and B), respectively. Yellow arrows indicate direction of cardiac conduction. Green circles indicate slow conduction nodes. Note the immature lateral conduction as well as heart block across ventricular myocardium (B and B′). (E and F) Immunolocalization of Cx40 and Cx43 in *sih* mutant hearts. Cx40 immunoreactivity was not observed in mutant hearts at 100 hpf. Atr, atrium; Ven, ventricle; AVC, AV canal.

**Figure 7:**
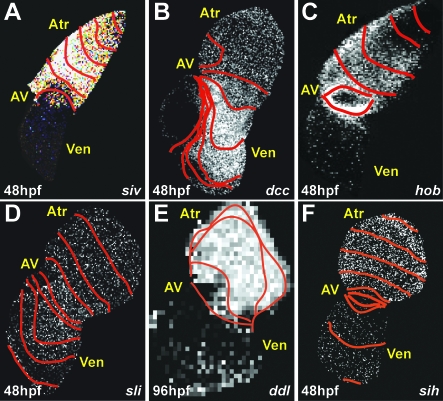
Optical Maps of Cardiac Conduction Mutants Isochronal lines indicate the distance of calcium activation waves traveled every 60 ms. (A) Ventricular conduction is absent in 48 hpf *siv* mutant hearts. (B) Disorganized ventricular conduction was observed in 48 hpf *dcc* mutant hearts. (C) AV conduction block is present in 48 hpf *hob* mutant hearts. (D) AV conduction delay is absent in 48 hpf *sli* mutant hearts. (E) Organized ventricular conduction is absent in 96 hpf *ddl* mutant hearts. (F) AV conduction delay is present in 48 hpf *sih* mutant hearts. Atr, atrium; AV, AV canal; Ven, ventricle.

Epigenetic factors, such as hemodynamic flow and cardiac contraction, previously have been suggested to influence the development of the CCS [6,9]. To determine the role of hemodynamic factors in the development of both slow/AV and fast/ventricular cardiac conduction pathways in zebrafish hearts, we performed optical mapping on the *silent heart* (*sih^b109^*) mutant heart, which fails to contract due to a null mutation in the *cardiac troponin T* (*tnnt2*) gene [15]. Optical mapping of 48 hpf *Tg*(*cmlc2*:*gCaMP*)*^s878^*; *sih* mutant hearts revealed an AV conduction delay, similar to that of 48 hpf wild-type hearts [5] (see Figure 7F). However, in contrast to the ventricular OC-to-base calcium activation pattern observed in wild-type 100 hpf hearts, 100 hpf *sih* mutant hearts displayed ventricular calcium activation laterally across the myocardium from the AV canal to the OFT as well as intermittent AV heart block (Figure 6A and 6B). Interestingly, this intermittent AV heart block occurred within the ventricle just after the AV canal (Figure 6B and 6B′). Similar conduction patterns also were observed in cardiac contractility mutants exhibiting weak contractions throughout the heart (unpublished data).

To determine possible etiologies for the conduction defects in *sih* mutant hearts, we analyzed rhodamine-phalloidin-stained *Tg*(*flk1*:*eGFP*)*^s843^*; *sih* mutants to assess the effects of the lack of blood flow and contraction on cardiac development. At 100 hpf, *sih* mutant hearts appear to have undergone cardiac looping and possess endocardium but do not exhibit trabeculae (Figure 6C). Additionally, 100 hpf *sih* mutant hearts showed significantly diminished Cx40 immunoreactivity but unaffected Cx43 immunoreactivity compared to wild type (Figure 6E and 6F). Thus, loss of hemodynamic blood flow and contraction results in the failure of the ventricle to develop trabeculae and the down-regulation of Cx40, thereby possibly affecting the development of the fast CCS.

### Forward Genetic Screen for Cardiac Conduction Mutants

We performed a large-scale ENU mutagenesis screen using the *Tg*(*cmlc2*:*gCaMP*)*^s878^* line as a secondary screen for physiologic analysis of cardiac conduction to identify genes that specifically regulate the development of the CCS. Intercrosses of F2 families were screened by visual inspection of live embryos for aberrant heart rates and/or coordination of atrial and ventricular contraction at each developmental stage of the CCS (24, 48, and 96 hpf). F2 carriers of putative mutations were outcrossed into the *Tg*(*cmlc2*:*gCaMP*)*^s878^* background to facilitate the physiologic analysis of cardiac conduction. Recovered mutations were organized into phenotypic groups and tested by complementation analysis. We identified 17 mutations defining 14 cardiac conduction regulating loci, four of which previously had been identified (Table 1). Physiologic analyses by optical mapping revealed that the identified mutations disrupt distinct developmental stages of the CCS. Representative examples of phenotypes observed are described below.

**Table 1:**
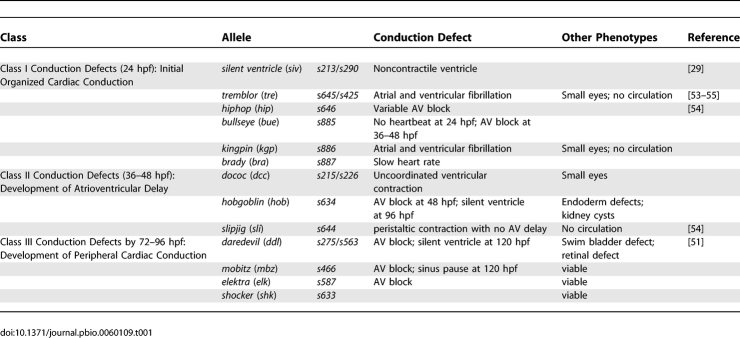
Cardiac Conduction/Rhythm Mutants

We recovered several noncontractile ventricle mutants including *s209*, *s264*, *s249*, *s271*, and *silent ventricle^s213^* ^,*s290*^ [28,29]. Previous work has suggested that a noncontracting ventricle phenotype may be due to contractile defects [15,19,30]. Utilizing the *Tg*(*cmlc2*:*gCaMP*)*^s878^*, we discovered that the *silent ventricle* (*siv*) mutant heart fails to generate cardiac conduction across the ventricular myocardium (Figure 7A). Further electrophysiologic characterization of *siv* mutant hearts revealed that the ventricular membrane potential was depolarized markedly. Hyperpolarization of mutant ventricle in current clamp mode resulted in spontaneous AP generation, restoring contractility [29].

We identified several mutants that had conduction defects across the AV myocardium. The *hob^ s634^* mutants develop AV heart block despite displaying a wild-type cardiac morphology and contractility (Figure 7C). However, *dococ^s215, 226^* (*dcc*) mutants display an uncoordinated contraction of the ventricle at 36–48 hpf, which was initially characterized by brightfield microscopy as ventricular fibrillation. However, optical mapping of 48 hpf *Tg*(*cmlc2*:*gCaMP*)*^s878^*; *dcc* mutants revealed that the AV conduction was blocked at the superior portion of the AV myocardium, resulting in heterogeneous and disorganized conduction across the ventricular myocardium (Figure 7B). In contrast, endocardial mutants including *cloche* [5,31], *santa*, and *valentine* [32] fail to display an AV conduction delay (unpublished data). Interestingly, the *slipjig^s644^* (*sli*) mutant, which has an endocardium and vasculature (unpublished data), also lacks an AV conduction delay (Figure 7D).

Finally, we identified a class of cardiac mutants that loses its ventricular conduction at 96 hpf. The mutant, *daredevil* (*ddl*), was initially characterized as a cardiovascular contractile mutant in which the ventricle became noncontractile by 96 hpf. Optical mapping of *Tg*(*cmlc2*:*gCaMP*)*^s878^*; *ddl* mutants revealed that these hearts developed heart block between 80–96 hpf and eventually lost all organized ventricular conduction by 96 hpf, resulting in a noncontractile ventricle (Figure 7E). Interestingly, these particular conduction mutants manifest their cardiac phenotype at the developmental stage when the fast CCS develops, suggesting that the affected genes may specifically regulate the development of the fast CCS.

**Figure 8:**
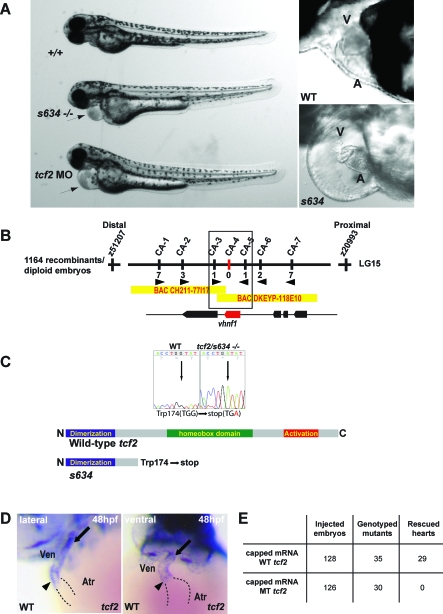
*s634* Affects *tcf2* (A) Lateral views of brightfield images of wild-type, *s634* mutant, and 0.5 ng *tcf2* ATG MO-injected embryos at 48 hpf, anterior to the left. Despite wild-type cardiac morphology, *s634* mutants and *tcf2* MO- injected hearts exhibit an AV conduction block, resulting in reduced cardiac output and pronounced pericardial edema (arrow). (B) Genetic map of the *s634* region. Numbers below simple sequence length polymorphism markers indicate recombination events. Two genes were identified within the critical region, which spans two bacterial artificial chromosomes. (C) Sequencing of *tcf2^s634^* cDNA revealed a G to A change at base pair 522, resulting in a premature stop codon at amino acid 174 thereby removing the homeobox and transactivation domains. (D) Whole mount RNA in situ hybridization at 48 hpf reveals *tcf2* expression at the AV canal (arrowhead) and the OFT (arrow) region of the ventricle. (E) Injection of wild-type *tcf2* mRNA rescued the heart phenotype of ∼82% of *s634* mutants, while injection of mutant *tcf2* mRNA failed to rescue. A, atrium; V, ventricle.

Because of its resemblance to human AV heart block (Figure 8A and Video S4), we isolated the gene disrupted by the *hob* mutation. Fine mapping of 1164 diploid embryos positioned the *hob* gene to a genomic interval of ∼150 kb on LG15 (Figure 8B). Sequence analysis of *tcf2* within the critical region revealed a G to A base pair change at position 552, leading to a premature stop codon after the dimerization domain thereby removing the homeobox and transactivation domains (Figure 8C). Data from morpholino (MO) knockdown, in situ analysis, and mRNA rescue experiments support the claim that *tcf2* is the gene affected by the *hob* mutation. Injection of 0.5 ng of *tcf2* MO recapitulated the *hob* mutant cardiac phenotype (Figure 8A). Whole mount in situ analyses reveal that *tcf2* is expressed at 48 hpf around the AV canal as well as the OFT portion of the ventricle (Figure 8D). Finally, injection of wild-type *tcf2* mRNA rescued the heart phenotype in ∼82% of *hob* mutants, while injection of mutant *tcf2* mRNA failed to rescue (Figure 8E). Altogether, these data indicate that *tcf2* contributes to the regulation of the cardiac conduction between the atrium and the ventricle.

## Discussion

**Figure 9:**
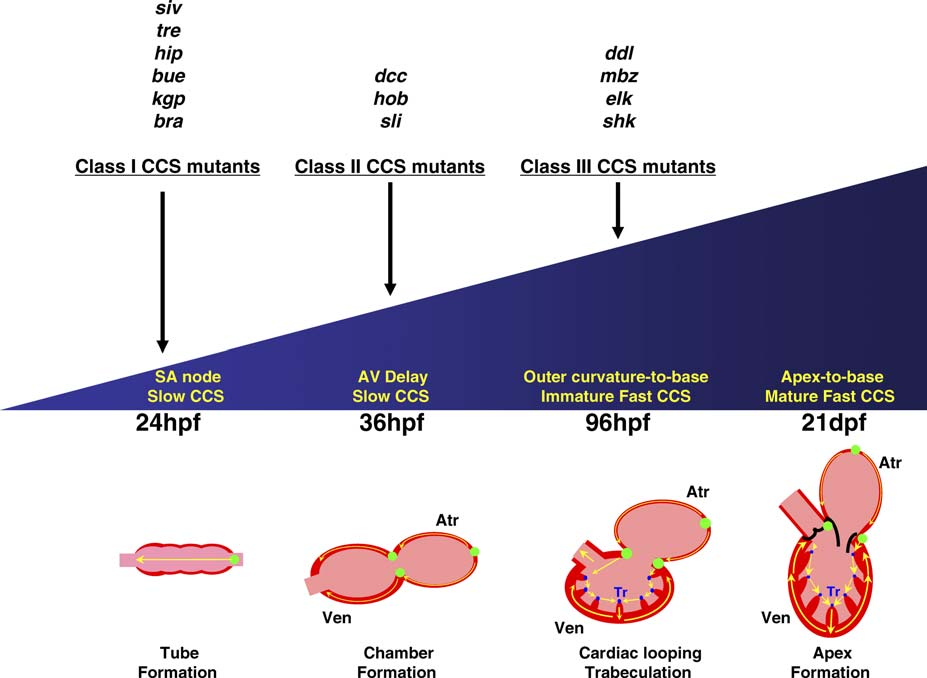
Development of the CCS and Classification of Mutants The diagram illustrates the four developmental stages of the CCS. Mutations affecting each stage are listed accordingly. Yellow arrows indicate direction of cardiac conduction. Green circles indicate slow conduction pathway/pacemaker and AV conduction delay. Blue circles indicate fast conduction network. Atr, atrium; Ven, ventricle; Tr, trabeculae.

Previous studies have utilized calcium green, a calcium-sensitive fluorescent dye, in zebrafish hearts to observe cardiac conduction up to 48 hpf [5,15]. Because of its short-lived expression, this method is inadequate to analyze later stages of CCS development. Thus, we comprehensively analyzed the development of the zebrafish CCS through cellular and physiologic studies using two new reporter lines: (1) *Tg*(*cmlc2*:*gCaMP*)*^s878^*, a myocardial-specific line that expresses the fluorescent calcium indicator protein, gCaMP, throughout cardiac development, and (2) *Tg*(*cmlc2*:*eGFP-ras*)*^s883^*, another myocardial-specific line that allows the morphological analysis of individual cardiomyocytes. These tools enabled us to describe four distinct physiologic cardiac conduction stages that correspond to distinct cellular and anatomical changes of the developing zebrafish heart (Figure 9). Furthermore, using this new in vivo optical mapping technique, we performed a physiology-based ENU mutagenesis screen to identify cardiac conduction phenotypes that would have escaped discovery or lacked sufficient characterization in earlier screens.

### Developmental Stages of the CCS

The vertebrate CCS can be divided into the slow conduction pathway, which regulates SA pacemaker activity and central AV conduction delay, and the fast conduction pathway, which allows apex-to-base conduction [33]. Through optical mapping of developing wild-type hearts, we observed distinct developmental stages for each of these CCS processes.

As early as the LHT stage, we observed that conduction propagates unidirectionally across the myocardium, suggesting the presence of SA node pacemaker activity [34]. Despite chamber specification, no significant conduction delay was detected at this stage. However, increased conduction velocity was observed in the ventricular half of the heart, suggesting that these cardiomyocytes have initiated cellular and molecular changes including the acquisition of relatively faster conducting properties. Interestingly, expression of *natriuretic peptide precursor a*, a marker of the OC and faster conduction velocities, recently has been observed in a subdomain of the ventricular portion of the LHT near the OFT [19].

The AV conduction delay was observed at 36–48 hpf, a time corresponding to the formation of the AV canal. Action potential and calcium transients of atrial, ventricular, and AV canal regions revealed electrophysiologic differences, suggesting that AV myocardial cells are distinct from atrial and ventricular cells. Whether these differences are due to unique channel expression versus distinct combinatorial effects of several cardiac channels remains unclear. However, weak expression of Connexin40, a marker for fast conduction, was observed at 48 hpf within the atrium and ventricle but lacking from the AV canal. In addition, the AV cardiomyocytes positioned themselves circumferentially around the AV canal. This ring-like orientation of cardiomyocytes has been suggested to contribute to the AV conduction delay [35].

Previous findings suggest that AV endocardial-specific signals, Neuregulin and Notch1b, cause the overlying AV myocardium to differentiate into slow conducting myocardium [5]. Additionally, *Tbx3*, a transcriptional repressor expressed in the developing conduction system in mouse [36], has been observed in the AV myocardium of 48 hpf zebrafish hearts [37]. Future studies exploring how these myocardial and endocardial factors may impact AV cardiomyocyte morphology, orientation, and action potential properties will be of great interest toward understanding the development of the AV conduction delay. Overall, our findings support studies suggesting that AV cardiomyocytes actively differentiate to become precursors of the AV node [5,20,38].

Despite lacking a distinct interventricular septum, adult zebrafish hearts possess a functionally fast CCS, resulting in an apex-to-base conduction across the ventricular myocardium [39]. Previous studies in mouse and chick have suggested that ventricular trabeculae and Cx40 may be responsible for an apex-to-base conduction prior to ventricular septation [6,21,40]. Consistent with these results, the rapid cardiac conduction network within the zebrafish ventricle develops as early as 96 hpf, a stage when cardiac looping is completed, trabeculae have started forming, and Cx40 expression is present. Loss of blood flow and cardiac contraction prevented the transition of ventricular conduction from a primitive linear pattern to a mature apex-to-base propagation, as previously observed in chick [6]. The absence of this transformation correlated with the lack of trabeculae and Cx40 immunoreactivity. Furthermore, we observed that *sih*/*tnnt2* mutants as well as weak cardiac contractility mutants also developed intermittent AV heart block below the AV canal, suggesting that this defect is within the proximal ventricular portion of the fast CCS rather than the slow AV conduction pathway. How these epigenetic factors modulate the development of the fast CCS remains unknown. However, similar to findings in chick, endocardial signals may be involved, as *cloche* mutant hearts also fail to develop trabeculae and a fast cardiac conduction network (unpublished data). Finally, a mature apex-to-base ventricular conduction was observed at 21 days postfertilization (dpf) when the hearts develop a distinct apex with increased ventricular trabeculation. Altogether, these data suggest that there is an intermediate step during the development of the mature ventricular apex-to-base conduction prior to the formation of the apex.

Overall, these cellular and electrophysiologic data provide the framework for future studies in zebrafish regarding the origin of the vertebrate CCS. Through lineage tracing and optical mapping studies, one should be able to address recent observations related to the ontogeny of the CCS from progenitors that also give rise to chambered myocardium [41].

### Systematic Analysis of the Identified Mutants Will Help Elucidate Mechanisms of Cardiac Conduction Development and Cardiac Arrhythmias

Previously, a candidate gene approach in zebrafish was performed to identify AV endocardial factors, *neuregulin* and *notch1b*, as regulators of AV conduction development [5]. In contrast, we have taken an unbiased approach and performed a new physiology-based forward genetic screen to identify additional mediators of the CCS. The recovered mutants display a wide spectrum of conduction phenotypes that first appear at distinct developmental stages of the CCS (Table 1 and Figure 9). Positional cloning reveals that *hob*, an AV conduction block mutation, affects the homeobox transcription factor, *Tcf2*. Given the increased prevalence of AV block in humans with type II diabetes mellitus independent of congestive heart failure and coronary artery disease [42], *Tcf2*, a mature onset diabetes of the young/diabetes-associated gene, may also directly mediate the development and maintenance of mammalian AV conduction. Interestingly, Tcf2 is known to regulate expression of *Na^+^*/*K^+^*-*ATPase* [43], an ion channel gene necessary for cardiomyocyte electrical polarization. Cardiac glycosides, such as digitalis, which inhibit the Na^+^/K^+^-ATPase pump, also can lead to human AV conduction heart block [44]. Additionally, mouse *Tcf2* in situ hybridization and real-time PCR analyses reveal its expression in the mammalian heart (Bussen M, Martin G, unpublished data). Thus, this mutant may help to address primary cardiac risk factors for a form of hereditary diabetes from indirect effects of diabetes on heart function.

Future analysis and isolation of the genes affected by these mutations promise to uncover new molecular clues and mechanisms that underlie both genetic and acquired forms of heart disease in humans. Novel genes discovered from this screen may be potential candidates for sequencing in population genetic studies of human cardiac arrhythmias. Conversely, candidate genes identified from human genome-wide association studies for cardiac arrhythmias may be validated rapidly and characterized by employing this in vivo optical mapping technique with MO knockdown experiments. Together, these studies will help develop therapeutic options aimed at maintaining and/or improving overall cardiac conduction.

## Materials and Methods

### Zebrafish husbandry and generation of the *Tg*(*cmlc2*:*gCaMP*)*^s878^* line.

Zebrafish were raised under standard laboratory conditions at 28 °C. We used the following transgenic lines: *Tg*(*flk1*:*EGFP*)*^s843^* [22] and *Tg*(*cmlc2*:*eGFP-ras*)*^s883^* (Jungblut B, Munson C, Huisken J, Trinh L, Stainier D, unpublished data). We generated the *Tg*(*cmlc2*:*gCaMP*)*^s878^* construct by cloning a 900 bp fragment of the *cmlc2* promoter [17] upstream of a promoter-less *gCaMP* construct [16]. We injected 200 pg of linearized DNA into one-cell-stage embryos and selected individual transgenic carrier adults by screening for fluorescent progeny. Six *Tg*(*cmlc2*:*gCaMP*) founders were recovered with identical expression patterns and levels. Homozygous mutant embryos were obtained by incrossing *Tg*(*flk1*:*EGFP*)*^s84^*/+; *sih*/+, *Tg*(*cmlc2*:*gCaMP*)*^s87^*/+; *sih*/+ [15], and *Tg*(*cmlc2*:*gCaMP*)*^s87^*/+; conduction mutants double heterozygotes.

### Immunohistochemistry, confocal microscopy, cell morphology, and in situ analysis.

Immunohistochemistry and confocal microscopy were performed as previously described [28,45,46]. The following antibodies were used at the following dilutions: rabbit polyclonal anti-Cx43 (Sigma) at 1:100 and rabbit polyclonal anti-Cx40 (Sigma) at 1:100. Cardiomyocyte surfaces and cross-sections were analyzed using ImageJ software (National Institutes of Health (NIH), http://rsb.info.hin.gov/ij/) as described previously [19]. A total of 288 cardiomyocytes were measured from 15 wild-type embryos: 165 OC, 83 IC, and 40 AV canal cardioymyocytes. Circularity measurements discriminate circular cells from elliptical cells. In situ analysis was performed as described [47].

### ENU mutagenesis and screen.

We screened approximately 9,076 F3 clutches from 2,392 ENU-mutagenized F2 families that were generated in the context of two different screens [28,48–51]. On the basis of the number of crosses per F2 family, we estimate that the screens surveyed 2,723 genomes. The specific locus test for each screen indicated a mutation rate of approximately 0.3% per gene per genome.

### Positional cloning.

Utilizing a set of simple sequence length polymorphism markers, we mapped *hob^s634^* to linkage group 5. Fine mapping with 1,164 mutant embryos narrowed the critical region to two bacterial artificial chromosomes: CHORI-211 77I17 and DanioKey pilot 118E10. This region contained two putative open reading frames, *tcf2*/*vhnf*-*1* and *gamma*-*synergin*. The *s634* cDNA was isolated, sequenced, and analyzed for both genes. A point mutation was discovered in *tcf2* and confirmed by sequencing *s634* genomic DNA. Approximately 0.5–1 ng of an ATG MO against *tcf2* (Gene-Tools), 5′-CTAGAGAGGGAAATGCGGTATTGTG-3′, was injected into one-cell-stage embryos. For mRNA rescue experiments, one-cell-stage embryos were injected with 50–100 pg of *tcf2* mRNA.

### Optical mapping by widefield epifluorescence.

Individual zebrafish between 24 hpf and 21 dpf were placed on a coverglass. Electromechanical isolation was achieved with 10 mM 2,3-butanedione monoxime (Sigma) applied 15 min before imaging. Single-plane widefield epifluorescence images of the heart were obtained with a Nikon TE-2000 inverted microscope, using 20× and 40× Plan Apo air objectives, an Xcite-120 (Exfo) widefield epifluorescent source, and standard fluorescein isothiocyanate filter set. Images were acquired with a Coolsnap HQ camera (Photometrics) using Metavue software (Molecular Devices) in stream acquisition mode at a frame rate of 30 ms/frame (512 × 512 pixels) for 48 hpf hearts and 15 ms/frame (256 × 256 pixels) for 96 hpf hearts. Image processing first consisted of manual adjustment of minor spatial shifts of the image over a temporal imaging series. Then, the fluorescence intensity of each pixel in a 2-D map was normalized to its percentage between the minimum and the maximum recorded values of the pixel over the full series. Isochronal lines at 60 ms intervals were obtained by identifying the maximal spatial gradient for a given time point. The color-coded scheme in each panel and video describes progressive activation of the heart with white/red cells and black/blue cells indicating depolarization and repolarization, respectively. Software processing was performed with Metavue software and procedures written in Matlab (Mathworks).

### Isolated calcium transient recordings by selective plane illumination microscopy.

Videos of the cardiac conduction wave were recorded with selective plane illumination microscopy [52]. The attenuated 488-nm laser line from a diode-pumped solid-state laser (Coherent Sapphire, 30 mW) was focused to a light sheet with a thickness of 6 μm. The sample was oriented such that a thin slice of atrium, AV canal, and ventricle was illuminated. The fluorescence was collected at 90 frames per second with a 20×/0.5 objective lens (Leica) and an emission filter (Chroma, HQ 525/50m) and imaged on an EM-CCD camera (Andor DV885). The microscope and camera were controlled with a Labview (National Instruments) program and analyzed with Matlab. In each sequence, several 15 × 15 pixel areas (i.e. 12 × 12 μm) were selected, and the intensity in these areas was plotted over time.

### Action potential recordings from embryonic heart.

The 48 hpf embryos were dechorionated and anesthetized with 0.02% tricaine for 1–2 min. The heart was dissected from the thorax en bloc, and all experiments were performed at room temperature. The recording chamber was perfused with the following solution containing (in mM) 140 NaCl, 4 KCl, 1.8 CaCl_2_, 1 MgCl_2_, 10 glucose, and 10 HEPES, pH 7.4. Suction pipettes were made from borosilicate capillary tubes (8250 glass, A-M Systems) and fire-polished to obtain resistances of 6–9 MΩ when filled with (in mM) 120 KCl, 5 EGTA, 5 K_2_ATP, 5 MgCl_2_, and 10 HEPES, pH 7.2. Transmembrane potential was measured using an Axoclamp 2A amplifier (Molecular Devices) in the bridge mode using the disrupted patch technique. The pipette was positioned adjacent to the heart, and a seal was formed by application of minimal suction. Through the use of this technique, stable spontaneous APs were recordable for up to 2 h. Transmembrane voltage was filtered at 10 kHz and digitized at a sampling frequency of 20 kHz with a 12-bit analog-to-digital converter (Digidata 1322A Interface, Molecular Devices).

## Supporting Information

Figure S1 Comparison of Calcium-Green–Injected Embryos versus Stable *Tg*(*cmlc2*:*gCaMP*)*^s878^* Embryos (A, E, I, M, Q) Epifluorescence micrographs of calcium-green-injected live zebrafish embryos at 24, 48, 72, 96, and 120 hpf. Note ubiquitous and strong fluorescence throughout most of the embryo.
(B, F, J, N, R) Higher magnification epifluorescence micrographs of calcium-green-injected embryos focusing on the hearts at 24, 48, 72, 96, and 120 hpf. Weaker calcium-green fluorescence is observed as the embryos develop.
(C, G, K, O, S) Epifluorescence micrographs of *Tg*(*cmlc2*:*gCaMP*)*^s878^* live embryos at 24, 48, 72, 96, and 120 hpf. Specific gCaMP fluorescence is detected only in hearts. Autofluorescence is detected in the yolk.
(D, H, L, P, T) Higher magnification epifluorescence micrographs of *Tg*(*cmlc2*:*gCaMP*)*^s878^* embryos focusing on the hearts at 24, 48, 72, 96, and 120 hpf. Autofluorescence from yolk does not interfere with imaging the hearts. At, atrium; V, ventricle; HT, heart tube.
(2.1 MB AI).

Video S1 Optical Mapping of 24 hpf *Tg*(*cmlc2*:*gCaMP*)*^s878^* Hearts Reveals Linear and Slow Conduction throughout the Heart Tube Calcium activation initiates at the sinus venosus pole (right) and travels across the heart tube to the OFT (left).
(3.4 MB MOV)

Video S2 Optical Mapping of 48 hpf *Tg*(*cmlc2*:*gCaMP*)*^s878^* Hearts Reveals AV Conduction Delay Calcium activation initiates in the atrium (upper right) and travels to the AV canal where it is significantly delayed. Calcium excitation is completed as the wave front propagates from the AV canal through the ventricle (lower left) and to the OFT.
(4.5 MB MOV)

Video S3 Optical Mapping of 100 hpf *Tg*(*cmlc2*:*gCaMP*)*^s878^* Hearts Reveals the Fast CCS Calcium activation initiates in the atrium (left) and travels to the AV canal where it is significantly delayed. Calcium excitation within the ventricle (right) initiates within the trabeculae and travels across the ventricular myocardium from the OC toward the base.
(5.4 MB MOV)

Video S4 48 hpf *hob^s634^* Mutant Heart Brightfield video of *hob^s634^* mutant heart. The 48 hpf *hob* mutant hearts develop AV heart block when heart chambers form. Not all atrial beats are conducted to the ventricle.
(4.4 MB MOV)

## Abbreviations

AP: action potential
AV: atrioventricular
CCS: cardiac conduction system
dpf: days postfertilization
ENU: ethylnitrosourea
gCaMP: a genetically encoded calcium reporter based on circular permutation of GFP
GFP: green fluorescent protein
hpf: hours postfertilization
IC: inner curvature
LHT: linear heart tube
MO: morpholino
OC: outer curvature
OFT: outflow tract
SA: sino-atrial
