# Supplementary material for: Genetic and Physiologic Dissection of the Vertebrate Cardiac Conduction System
Source: PLoS Biol. 2008 May 13;6(5):e109. doi: 10.1371/journal.pbio.0060109 (PMC2430899; doi:10.1371/journal.pbio.0060109)
Supplement: Figure S1 — (A, E, I, M, Q) Epifluorescence micrographs of calcium-green-injected live zebrafish embryos at 24, 48, 72, 96, and 120 hpf. Note ubiquitous and strong fluorescence throughout most of the embryo. (B, F, J, N, R) Higher magnification epifluorescence micrographs of calcium-green-injected embryos focusing on the hearts at 24, 48, 72, 96, and 120 hpf. Weaker calcium-green fluorescence is observed as the embryos develop. (C, G, K, O, S) Epifluorescence micrographs of Tg(cmlc2:gCaMP)s878 live embryos at 24, 48, 72, 96, and 120 hpf. Specific gCaMP fluorescence is detected only in hearts. Autofluorescence is detected in the yolk. (D, H, L, P, T) Higher magnification epifluorescence micrographs of Tg(cmlc2:gCaMP)s878 embryos focusing on the hearts at 24, 48, 72, 96, and 120 hpf. Autofluorescence from yolk does not interfere with imaging the hearts. At, atrium; V, ventricle; HT, heart tube. (2.1 MB AI). [file pbio.0060109.sg001.pdf]

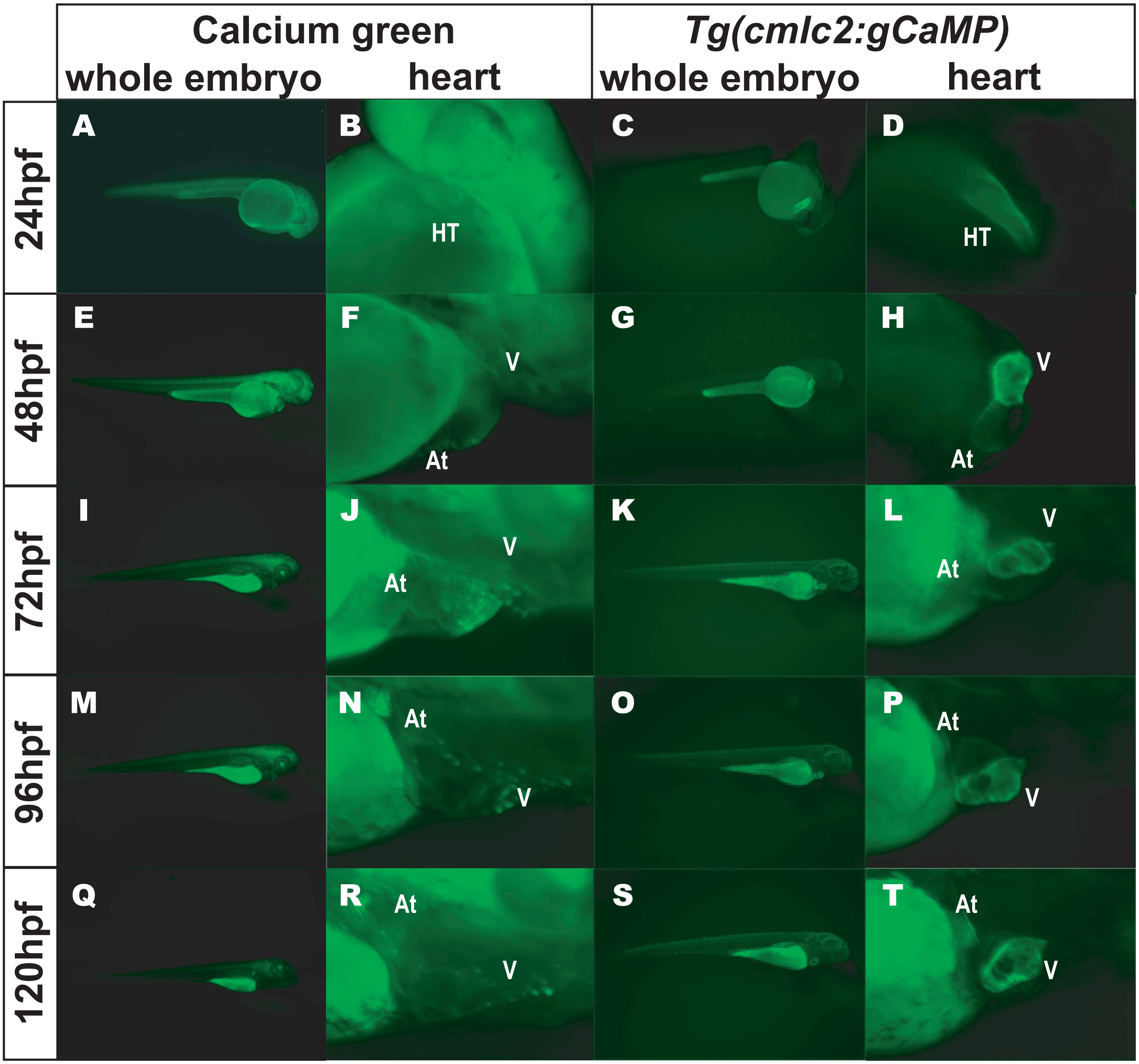

Calcium green
whole embryo
heart
Tg(cmlc2:gCaMP)
whole embryo
heart
24hpf
48hpf
72hpf
96hpf
120hpf
A
B
HT
C
D
HT
E
F
V
At
G
H
V
At
I
J
V
At
K
L
V
At
M
N
At
V
O
P
At
V
Q
R
At
V
S
T
At
V
